# Antimicrobial use and resistance in food-producing animals and the environment: an African perspective

**DOI:** 10.1186/s13756-020-0697-x

**Published:** 2020-03-03

**Authors:** Zuhura I. Kimera, Stephen E. Mshana, Mark M. Rweyemamu, Leonard E. G. Mboera, Mecky I. N. Matee

**Affiliations:** 1 Department of Microbiology and Immunology, School of Medicine, Muhimbili University of Health and Allied Sciences, Dar es Salaam, Tanzania; 2 Ministry of Livestock and Fisheries, Dodoma, Tanzania; 3 Department of Microbiology and Immunology, Catholic University of Health and Allied Sciences, Mwanza, Tanzania; 4 SACIDS Foundation for One Health, Sokoine University of Agriculture, Chuo Kikuu Morogoro, Tanzania

**Keywords:** Antimicrobial use, Antimicrobial resistance, Surveillance, Food animals, Environment, Africa

## Abstract

**Background:**

The overuse of antimicrobials in food animals and the subsequent contamination of the environment have been associated with development and spread of antimicrobial resistance. This review presents information on antimicrobial use, resistance and status of surveillance systems in food animals and the environment in Africa.

**Methods:**

Information was searched through PubMed, Google Scholar, Web of Science, and African Journal Online databases. Full-length original research and review articles on antimicrobial use, prevalence of AMR from Africa covering a period from 2005 to 2018 were examined. The articles were scrutinized to extract information on the antimicrobial use, resistance and surveillance systems.

**Results:**

A total of 200 articles were recovered. Of these, 176 studies were included in the review while 24 articles were excluded because they were not relevant to antimicrobial use and/or resistance in food animals and the environment. The percentage of farms using antimicrobials in animal production ranged from 77.6% in Nigeria to 100% in Tanzania, Cameroon, Zambia, Ghana and Egypt. The most antibiotics used were tetracycline, aminoglycoside and penicillin groups. The percentage of multi drug resistant isolates ranged from 20% in Nigeria to 100% in South Africa, Zimbabwe and Tunisia. In the environment, percentage of multi drug resistant isolates ranged from 33.3% in South Africa to 100% in Algeria. None of the countries documented national antimicrobial use and resistance surveillance system in animals.

**Conclusion:**

There is high level of antimicrobial use, especially tetracycline, aminoglycoside and penicillin in animal production systems in Africa. This is likely to escalate the already high prevalence of antimicrobial resistance and multi drug resistance in the continent. This, coupled with weak antimicrobial resistance surveillance systems in the region is a great concern to the animals, environment and humans as well.

## Introduction

Food animals such as cattle, poultry and pigs have been extensively reared worldwide, not only as a source of food but also a source of income. The modes of productions are intensive [1], due to the rapidly increasing demand for livestock products driven by human population growth and urbanization [2, 3]. This has necessitated the uncontrolled use of antimicrobials [4], which has been associated with increase of antimicrobial resistance [5].

The subsequent contamination of soil, sediments, sludge, groundwater, wastewater, tap and surface water, and plants contribute to the emergence and spread of multi-drug (MDR) organisms in environment [6, 7]. Furthermore, unmonitored quantities of waste that contain antimicrobials generated by pharmaceutical manufacturers, hospitals, and livestock producers promote selection of resistomes in the environment, with potential spill over to animals and humans [8, 9]. Some of important organisms that have been found to circulate in different compartments include *Campylobacter* spp., *Salmonella* spp., *Staphylococcus* spp., *Enterococcus* spp. and ESBL- producing *Enterobacteriaceae* [10–12].

Although AMR is a global threat [13], the situation in Africa is compounded by a number of factors that include lack of access to appropriate antimicrobial therapy, weak of regulation in the use of antimicrobials for human and animal, weak surveillance systems, lack of updated antimicrobial use and treatment guidelines. Others are lack of continuing education on antimicrobial use (AMU) for prescribers, tendency for animal owners to stock drugs and engaging unskilled people to treat animals, high degree of drugs abuse by livestock keepers and unregulated disposal of waste in dumps [14–17]**.**

Despite these facts, there is paucity of consolidated information on the antimicrobial use (AMU), antimicrobial resistance (AMR), surveillance and stewardship programmes in Africa. The few available reviews on antimicrobial use and resistance are mostly country specific [18, 19]. This study reviewed the use of antimicrobial agents, prevalence of antimicrobial resistance and status of surveillance systems in food-producing animals and the environment in Africa.

## Methods

This review was carried out between October 2018 and April 2019. Pub-Med, Google Scholar, Web of Science, Africa Wide Information and African Journal Online databases were searched for information on AMU and AMR covering a period from 2005 to 2018. Full-length research articles and review papers written in English were considered. In addition, publications from Food and Agriculture Organization (FAO), World Health Organization (WHO), International Livestock Research Institute (ILRI), Office International des Epizooties (OIE) websites were also searched and reviewed.

Combinations of search terms used were ‘antimicrobial usage’, ‘antimicrobial use’, ‘antibiotic use’, ‘antimicrobial resistance’, ‘antimicrobial resistant’, ‘food-producing animals’, ‘food animals’, animal husbandry’, ‘animal farming’, ‘domestic animal farming’, ‘farmed animals’, ‘environment’, ‘environmental’, ‘waste water’, treated waste water’, ‘sea water’, ‘river water’, ‘effluent’, ‘irrigation water’, ‘surface water’, ‘soil’ and ‘vegetables’. Others were specific food animal descriptors such as ‘poultry’, ‘chickens’, ‘pigs’, ‘swine’, ‘cattle’, ‘beef cattle’, ‘dairy cattle’, ‘fish’, specific country by name, and the word ‘Africa’. The articles were scrutinized to extract information on the antimicrobial use, prevalence of AMR and availability of a surveillance system.

## Results

**Fig. 1:**
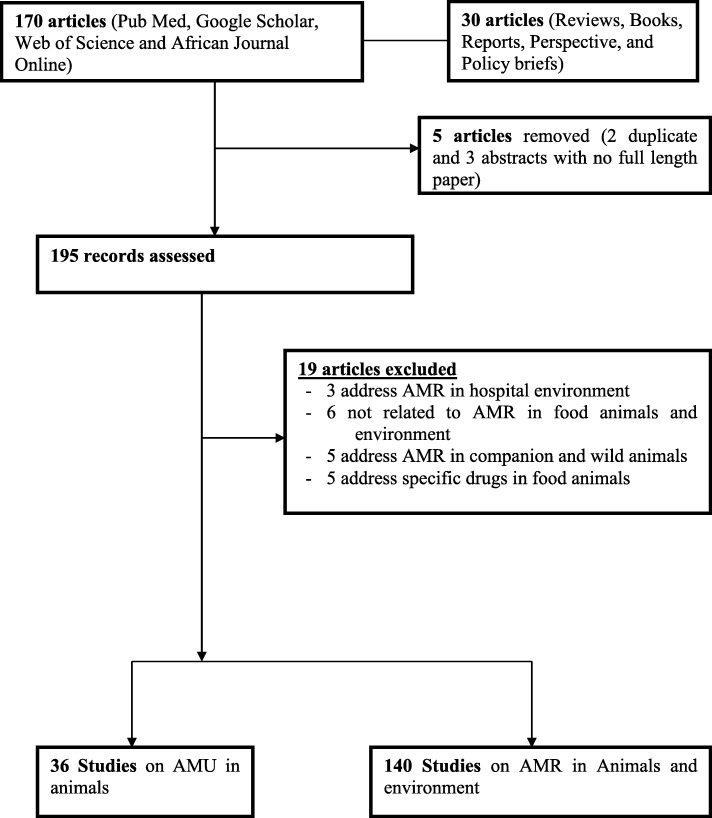
Flow diagram of number of articles obtained and those eliminated

A total of 200 articles were recovered, of which 170 were original research articles and 30 were reviews, books, reports and perspectives or policy briefs. On further evaluation, five were removed, of which two reported similar data and three were abstracts whose full length papers could not be accessed. Of the 195 articles assessed, 19 were excluded from review because they were not relevant (Fig. 1), therefore only 176 studies were included in this review.

### Types of study and geographical distribution

**Fig. 2:**
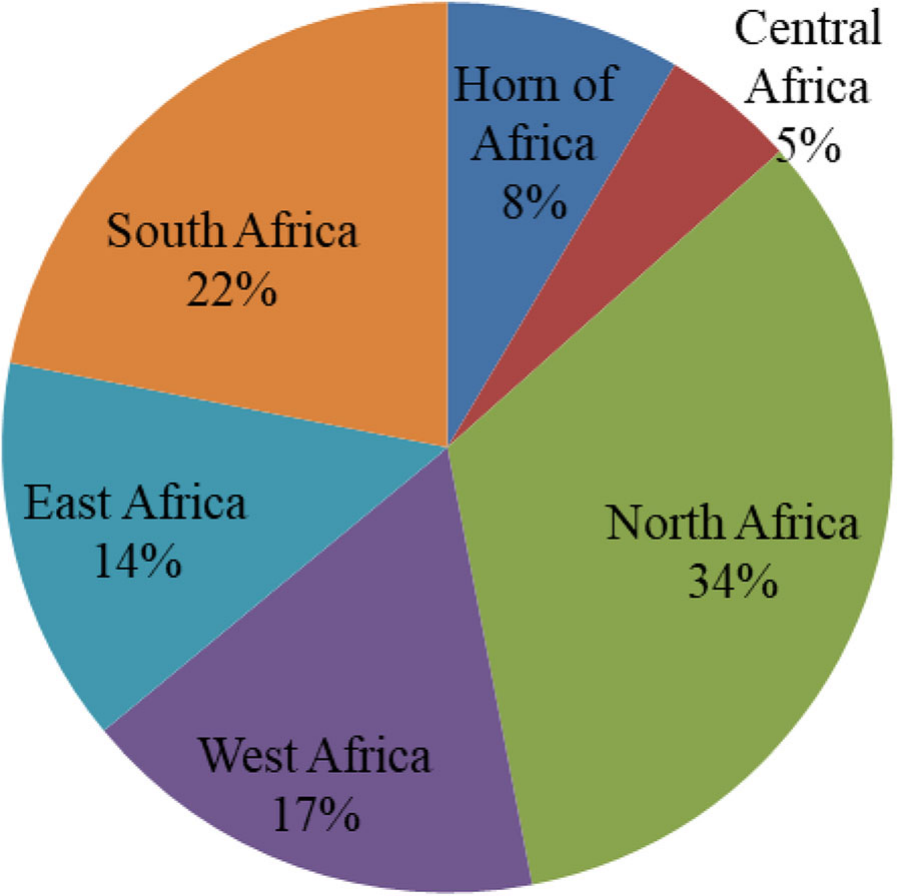
Geographical distribution of AMU and AMR studies in animal and environment

The majority of the studies were cross-sectional in design, with only two being retrospective (one presenting data on AMU and the other on AMR patterns). Only three studies quantified the amount of antimicrobial using the defined daily dose per animal (DDDA). Some studies involved both surveillance of antimicrobial use and laboratory analysis of organisms’ (Table 1). Geographically, most (56%) of the studies were from the northern and southern Africa regions (Fig. 2).

**Table 1:** Different study designs used in data collection on antimicrobial use and resistance

Sample type	Study type	Approach	Number of articles
Food animals	Cross sectional	Surveillance (Questionnaire, observation and/or focus group discussion and in-depth interview)	9
Food animals	Cross sectional	Laboratory analysis of antimicrobial susceptibility and resistant organisms	92
Food animals	Cross sectional	Laboratory and surveillance	14
Fish	Cross sectional	Laboratory analysis of the resistant organism(s)	7
Environment	Cross sectional	Laboratory analysis of the resistant organism(s)	26
Food animals and environment	Cross sectional	Surveillance with laboratory analysis	9
Food animals and environment	Retrospective	Surveillance and laboratory analysis of the resistant organisms	3

Food animals referred here includes all domestic animals farmed for food consumption (cattle, sheep, goats, poultry, camel, horse, rabbit and donkey); Fish involved those captured from natural water bodies (river, streams, dams and ocean) and the environment samples involved treated waste water, effluent, surface water, river and ocean water, sediments and soil

**Fig. 3:**
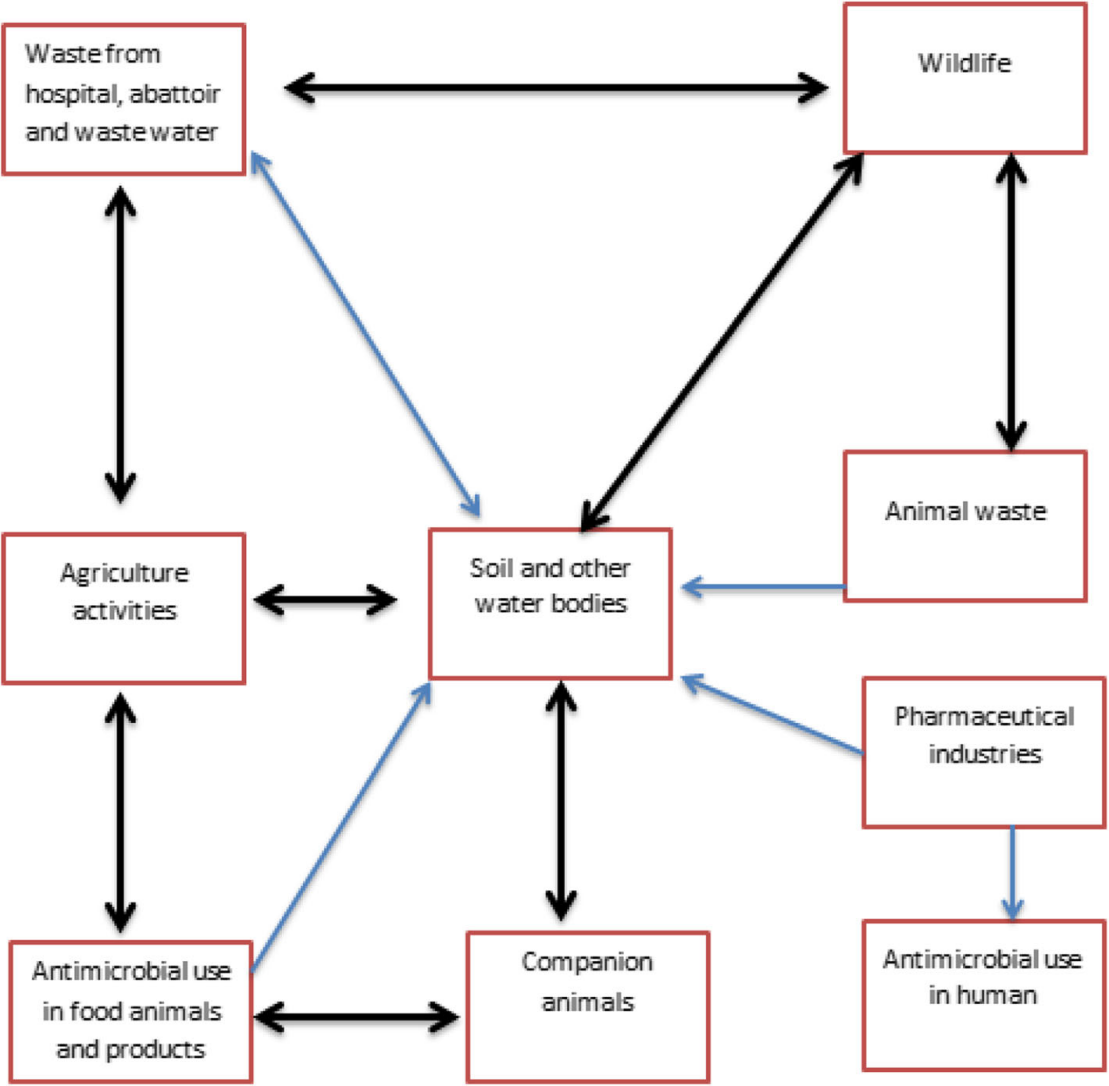
Complex interactions involved in the spread antimicrobial resistance between sectors

### Antimicrobial use in animals

As shown in Table 2, antimicrobial agents were administered to different food animal species, mostly in poultry. The percentage of farms using antimicrobial agents ranged from 77.6% in Nigeria to 100% in Tanzania, Cameroon, Zambia, Ghana and Egypt. In total 14 different classes of antimicrobial agents were used, mainly tetracycline, aminoglycosides and penicillin. Antimicrobial agents such as macrolides, which are restricted for use in animal in some developed countries due to their impact in human health, were used in Africa.

**Table 2:** Percentage of farms using antimicrobials by country, type of animal and class of antimicrobials

Country	Food animal	% AMU	Class of antimicrobial	Reference
Ghana	Poultry	98	Tetracyclines, Aminoglycosides, Penicillins, Quinolones	[20]
Ghana	Cattle, goat, sheep, pig, poultry	98	Tetracyclines, Penicillins, Macrolides, Aminoglycosides, Sulphonamides, Benzimidazoles	[21]
Tanzania	Cattle, chickens, pigs	100	Tetracyclines, Sulphonamides, Penicillins, Aminoglycosides	[22]
Cameroon	Poultry	100	Aminoglycosides, Sulphonamides, Quinolones, Macrolides, Tetracyclines, Penicillins	[23]
Sudan	Poultry	92	Tetracyclines	[24]
Nigeria	Cattle, sheep, goats	77.5	Tetracyclines, Quinolones, Penicillins, Aminoglycosides	[25]
Zambia	Cattle		Aminoglycosides, Sulphonamides, Macrolides, Penicillins, Polypeptides, Tetracyclines	[17]
Zambia	Cattle	100	Tetracyclines, Penicillins,	[26]
Tanzania	Cattle, goat, sheep, pigs, poultry	74	Tetracyclines, Penicillins, Macrolides, Aminoglycosides, Sulphonamides	[15]
Ghana	Pigs	100	Tetracyclines, Sulphonamides, Penicillins, Quinolones, Macrolides, Aminoglycosides	[27]
Tanzania	Cattle	85	Tetracyclines	[28]
Tanzania	Poultry	90	Tetracyclines, Sulphonamides, Dihydrofolate, Aminoglycosides, Quinolones	[14]
Sudan	Poultry, cattle, sheep, goats	95	Tetracyclines, Penicillins, Macrolides, Sulphonamides, Aminoglycosides, Lincosamides, Streptogramins, Quinolones	[29]
Ethiopia	Cattle, poultry	80	Tetracyclines, Penicillins, Sulphonamides	[30]
Nigeria	Poultry	88.5	Tetracyclines, Aminoglycosides, Macrolides, Quinolones, Penicillins, Sulphonamides, Furanes, Polypeptides	[31]
Uganda	Pigs	40.6	Dihydrofolate, Tetracyclines, Aminoglycosides,	[32]
Cameroon	Poultry	80	Tetracyclines, Macrolides, Phenocols, Aminoglycosides	[12]
Egypt	Poultry	100	Tetracyclines, Quinolones	[33]
Uganda	Poultry	96.7	Sulphonamides	[34]
Nigeria	Cattle	77.6	Tetracyclines, Macrolides, Penicillins, Aminoglycosides, Sulphonamides, Quinolones	[35]

### Antimicrobials use in the environment

Figure 3 below summarizes the complex interactions between human, animal husbandry, veterinary medicine and the environment with potential for occurrence and spread of AMR resistomes. Such complexity complicates the interventions involved in curbing the burden of AMR.

### Antimicrobial resistance in food animals

This review highlights different levels of antimicrobial resistance within and between countries. Proportion of MDR strains among *E. coli*, which is an indicator organism, is shown in Table 3. The multi-drug resistance is referred as non-susceptibility to more than three antimicrobial categories [54]. The prevalence of MDR *E.coli* ranged from 20% in Nigeria to 100% in South Africa, Zimbabwe and Tunisia, in most cases being above 80%. Of the 14 different classes of antimicrobial agents, higher prevalence of resistance was reported for tetracycline, sulphonamides and penicillin, which are the cheapest and therefore more widely used antibiotics.

**Table 3:** Proportion of MDR strains among *Escherichia coli* isolated from food animals

Sample type	% MDR	Antimicrobial class resisted	References
Poultry	92.6	Tetracycline, Penicillin, Quinolones, Phenocols, Sulphonamides, Cephalosporins	[36]
Cattle, goat, sheep, pig, poultry	91.6	Tetracycline, Penicillin, Phenocols, Aminoglycosides, Sulphonamides	[21]
Poultry	42.9	Tetracycline, Sulphonamides, Penicillin, Aminoglycosides, Quinolones, Phenocols, Cephalosporins	[37]
Poultry, pigs	20	Quinolones, Sulphonamides, Macrolides, Tetracycline, Phenocols, Penicillin, Aminoglycosides	[38]
Cattle	100	Phenocols, Penicillin, Tetracycline, Cephalosporins, Sulphonamides, Aminoglycosides, Quinolones	[39]
Poultry	62	Tetracycline, Quinolones, Sulphonamides	[40]
Cattle, pigs	93.4	Tetracycline, Sulphonamides, Macrolides	[41]
Cattle, pigs poultry	45.5	Tetracycline, Penicillin, Sulphonamides, Dihydrofolate, Penams, Macrolides, Cephalosporins, Clavam	[42]
Poultry	83	Tetracycline, Sulphonamides, Quinolones, Aminoglycosides, Dihydrofolate, Penicillin, Phenocols	[43]
Poultry	80	Quinolones, Tetracycline, Penicillin, Aminoglycosides, Sulphonamides, Phenocols	[44]
Poultry	100	Tetracycline, Penicillin, Quinolones, Aminoglycosides	[45]
Cattle, pigs, poultry	65.5	Tetracycline, Sulphonamides, Penicillin, Aminoglycosides, Quinolones, Clavam Glycopeptide, Cephalosporins, Dihydrofolate	[46]
Poultry	40	Tetracycline, Dihydrofolate, Phenocols Sulphonamides, Aminoglycosides	[47]
Fish	54.5	Cephalosporins, Aminoglycosides, Sulphonamides, Quinolones	[48]
Poultry	90	Penicillin, Clavam, Penams, Quinolones Cephalosporins, Aminoglycosides	[49]
Pigs	80	Penicillin, Penams, Tetracycline, Quinolones, Cephalosporins, Aminoglycosides	[50]
Poultry	80	Penicillin, Macrolides, Aminoglycosides, Tetracycline, Sulphonamides, Phenocols, Dihydrofolate, Quinolones	[33]
Poultry	100	Tetracycline, Quinolones, Dihydrofolate, Sulphonamides, Aminoglycosides	[51]
Poultry	65	Tetracycline, Penicillin, Sulphonamides, Phenocols	[52]
Fish	100	Aminoglycosides, Cephalosporins, Phenocols, Tetracycline, Sulphonamides, Dihydrofolate, Clavam, Penicillin	[53]

### Antimicrobial resistance in environment

In this review only a few studies reported AMR in the environment. These studies involved samples collected from domestic and biomedical waste, waste water, river sediments, surface and drinking water, treated waste water, river water and vegetables (Table 4). The prevalence of MDR *E. coli* in environmental samples ranged from 33.3% in South Africa to 100% in Algeria and South Africa. In total *E. coli* exhibited resistance to 16 different antimicrobial agents.

**Table 4:** Percentage of MDR *Escherichia coli* from environmental samples

Country	Sample type	%MDR	Antimicrobial class	References
South Africa	Treated Waste water	75.9	Lincosamides, Sulphonamides, Carbapenems, Quinolones, Penicillin, Tetracycline, Polypeptide, Dihydrofolate, Aminoglycosides, Cephalosporins Macrolides	[55]
South Africa	River water, Sediments	84	Furans, Penicillin, Clavam, Quinolones, Phenocols, Dihydrofolate, Cephalosporins	[56]
Ethiopia	Drinking water	66.7	Penicillin, Clavam, Quinolones, Cephalosporins, Tetracycline, Phenocols, Sulphonamides, Aminoglycosides	[57]
South Africa	River water	100	Penicillin, Tetracycline, Aminoglycosides, Cephalosporins, Quinolones, Dihydrofolate	[58]
South Africa	Treated waste water	33.3	Tetracycline, Penicillin, Furanes, Aminoglycosides, Cephalosporins, Phenocols, Quinolones, Polypeptide, Lipopeptides	[59]
Algeria	Treated waste water	85	Cephalosporins, Quinolones, Sulphonamides, Aminoglycosides, Tetracycline, Phenocols	[60]
Algeria	River water	100	Penicillin, Clavam, Monobactams	[61]
Egypt	River water	82.5	Penicillin, Glycopeptides, Macrolides, Lincosamides, Dihydrofolate, Tetracycline, Sulphonamides	[62]
Tunisia	Waste, Surface water	76	Aminoglycosides, Dihydrofolate, Quinolones, Sulphonamides, Phenocols, Tetracycline, Cephalosporins	[6]
Tanzania	Domestic, Biomedical waste, River sludge	56	Aminoglycosides, Cephalosporins, Quinolones, Penicillin	[63]

### Genetic relatedness of resistant genes in animals, humans and environment

We identified a few studies that had determined AMR in all three compartments, namely animals, humans and the environment. Although most of the studies involved phenotypic methods, few involved molecular characterizations as well. The most frequently used molecular methods were Polymerase Chain Reaction (PCR), Pulse Gel Field Electrophoresis (PGFE), and Multilocus Sequence Typing (MLST). A summary of AMR genes in the three compartments is shown in Table 5. Some genes such as *bla*_CTX-M_, *bla*_TEM_, *bla*_SHV,_ *bla*_OXA,_ *aac(6*′*)- Ib-cr, tet(A)*, *tet(B), sul1*, *sul2,* and *qnr* were commonly detected in all of the three compartments, indicating a potential flow of AMR genes across humans, animals and environment [64–67].

**Table 5:** A summary of resistant genes detected in humans, animals and environment

Methodology	AMR genes			Reference
	Human	Animals	Environment	
Disc diffusion, CHROMagar™, Etest, MALDI-TOF-MS, MLST, PFGE, PCR, Sequencing	bla_CTX-M-15_, bla_TEM-1_, bla_OXA-1_, aac(6′)- Ib-cr	bla_CTX-M-15_, bla_OXA-1_, aac(6′)- Ib-cr, bla_TEM-1_	bla_CTX-M-15_, bla_TEM-1_, bla_OXA-1_, aac(6′)- Ib-cr	[64]
Disk diffusion, PCR, sequencing	bla_TEM-1_, bla_OXA-10_, bla_CTX-M-15_	bla_TEM-1_, bla_TEM-57_		[65]
PRISMA guidelines	bla_CTX-M-15_	bla_CTX-M-15_	bla_CTX-M-15_	[66]
MacConkey agar, Disk diffusion, PCR	bla_KPC_, bla_OXA-48_, bla_NDM_	bla_KPC_, bla_OXA-48_, bla_NDM_	bla_KPC_, bla_OXA-48_, bla_NDM_	[67]
PRISMA guidelines	bla_SHV_, bla_TEM_, bla_Z_, tet(A), tet(B), tet(C), tet(K), vanA, vanB, vanC1, ermB, vanC2/3,erm, aac (6′)-aph (2″), aac(6′)-lb-cr, bla_GES_, bla_GIM_, bla_IMP_, bla_KPC_, bla_NDM_, bla_NDM-1_, bla_PER_, bla_SIM_, bla_SPM_, bla_VIM_, cfiA, cfxA, CjgyrA, CmeB, eis, embB, frxA, gidB, gyrA, gyrB, inhA, katG, mcr-1 plasmid, mtrR, mupA, nimA-J, parC, parE, penA, pilQ, pncA, ponA porB1b (penB), qepA, qnrA, qnrB, qnrC, qnrD, qnrS, rdxA, rpoB, Tet(O), tet(Q)	bla_SHV_, bla_TEM_, bla_Z_, tet(A), tet(B), tet(C), tet(K), vanA, vanB, vanC1, ermB,vanC2/3, erm, ant (3″)-la, ermA, mphC, msrA	bla_SHV_, bla_TEM_, bla_Z_, tet(A), tet(B), tet(C), tet(K), vanA, vanB, vanC1, ermB, vanC2/3, erm, aadA, aadA1, Bla, cat I, cat II, cmlA1, dfr18, dfrA1, dfrA1, floR, mefA, srC, strB, sul3, tet(D), tet(E), tet(G), tet(H), tet(J), tet(L), tet(Y)	[68]
MacConkey with 2 mg/L cefotaxime, Disc diffusion, VITEK®2 system and WGS		bla_CTX-M-15_, bla_TEM-1_, bla_OXA-1_, aac(6′)- Ib-cr, strA, strB, aac(3)-IId, aadA1, qnrB1, qnrS1, sul1, sul2, dfrA14, dfrA17,dfrA1,dfrA18, tet(A), dfrA30, dfrA5, dfrA7, tet(D), bla_SHV-11_, bla_SHV-1_, bla_ACT-15_, bla_MIR-3_, bla_CMY-37_, bla_CMY-49_, aadA2, aac(3)-IIa, qnrB29, qnrB48, oqxA, oqxB.	bla_CTX-M-15_, bla_TEM-1_, bla_OXA-1_, aac(6′)- Ib-cr, strA, strB, aac(3)-IId, aadA1, qnrB1, qnrS1, sul1, sul2, dfrA14, dfrA17, dfrA1, dfrA18, dfrA30, dfrA5, dfrA7, tet(A), tet(D), aadA5	[69]
MacConkey agar with 2 mg/mL cefotaxime, ChromAgar, Disk diffusion, WGS VITEK®2		bla_CTX-M-15_, strA, strB, aac(6)-Ib-cr, qnrS1		[70]
MacConkey agar with 2 mg/mL cefotaxime, Disk diffusion, VITEK®2, WGS CHROMagar,	bla_CTX-M-15_, aac(6′)Ib-cr, strA, strB, qnrS1			[71]
MacConkey broth, Disc diffusion, API 20E, PCR, Sequencing	Tet(B)	Tet(B)		[41]
Disk diffusion, MLST, WGS	strA, strB, aadA1, tet(B), bla_TEM-1B_, catA1, sul1, sul2, dfrA1	strA, strB, aadA1, tet(B), bla_TEM-1B_, catA1, sul1, sul2, dfrA1		[72]
API 20E System, MALDI-TOF, PCR	bla_CTX-M-1_, bla_CTX-M-15_, bla_TEM_	bla_CTX-M-1_, bla_TEM_		[73]
MacConkey and XLD agar, TSI, API 20 E, Disk diffusion, PCR		sul1 and tet(A)	sul1 and tet(A)	[74]
MacConkey with cefotaxime (2 mg/l), MCA with ciprofloxacin, double-disc synergy test, PCR, MALDI, MLST, PFGE	bla_TEM-1_, bla_CTX-M-15_, sul1, sul2, tet(B), catA1, oqxA, dfrA17-aadA5, qepA, strA, tet(A), dfr12-orf-aadA2, qnrS1, aadA1, strA-B, tet(D), oqxB, sul3, dfrA7, aadA5, dfr1-sat-aadA1, qnrB1, aac(60)Ib-cr, bla_OXA-1_, bla_SHV-2a_, bla_CTX-M variant 2_	bla_TEM-1_, bla_CTX-M-15_, sul1, sul2, tet(B), catA1, oqxA, dfrA17-aadA5, qepA, strA, tet(A), dfr12-orf-aadA2, qnrS1, aadA1, qnrB33, qnrB17, qnrB28, bla_SHV-62_		[75]
Slide agglutination, ETest, PCR	bla_TEM_, bla_SHV_, bla_CTX-M_, tet(A), floR, sul2, dfrA1, dfr18, strA, strB, qnrVC3, gyrA, gyrB		bla_TEM_, bla_SHV_, bla_CTX-M_, tet(A), floR, sul2, dfrA1, dfr18, strA, strB, qnrVC3, gyrA, gyrB	[76]

### Surveillance systems

Our review revealed that there were no country with a documented national AMU and AMR surveillance programme specific for either animals or environment. Most countries rely on point-prevalence rather than nation-wide surveillance programmes [19, 77]. Some countries have developed a national antimicrobial plan, as an initial stage towards developing AMU and AMR surveillance system in human, animal and environment. Fifteen countries have approved National action Plan for Antimicrobial resistance (NAP), while eight are waiting for the authority’s approval or they are heading towards finalization [78]. It was noted that there is support on capacity to develop surveillance and monitoring of AMU and AMR in food and agriculture provided by the World Health Organization in collaboration with Food and Agriculture Organization and World Organization for Animal Health (OIE) [79].

Other forms of supports included: (i) value chain analysis in animal health from farm to retail step, focusing on microbiology analysis [80]; (ii) training, technical support, strategic guidance and software tools for Web-based data entry and collaboration for the studies targeting animals, environment and other sectors [81]; (iii) training of laboratory technicians from human, agriculture and veterinary sectors from eight countries on laboratory surveillance and control of major food borne diseases [82]; and (iv) research projects on integrated surveillance of AMR in foodborne bacteria for Chad, Tanzania and Ethiopia [82]. Recently, the Africa Centre for Disease Control has launched a network called Antimicrobial Resistance and Surveillance Network comprising experts from animal, environmental and human health sectors. The network targets to mitigate harm from antimicrobial resistant organisms arising from animals, environment, agriculture and human [83]. According to WHO [82] other initiatives provided to countries in Africa included studies on ESBL-producing *E.coli* in animals and the environment organized in Egypt, Morocco and Sudan.

## Discussion

This review involved studies with different designs in evaluation of AMU and AMR. Such variations, especially in sampling designs, complicate comparison of the reported data, and may impact on the reliability of data [32, 78]. There are also potential challenges such as: financial constraints, skilled human resource, lack of research facilities and lack of awareness on the role of animals and environment in the spread of antimicrobial resistances that differ within and between countries. As a consequence, most of the reported studies are from northern Africa, where the issues of AMU and AMR could be less critical than in other parts of Africa.

The review has revealed that more than 80% of farms use antimicrobial agents in animal production, driven by the increase in demand for food of animal origin and emerging trade opportunities [2, 84]. Some of the antimicrobial agents used such as macrolides that have been restricted for use elsewhere due health risk concerns [33, 35] were commonly used in Africa. We found higher AMR prevalence in tetracycline, aminoglycosides and penicillin, these are the cheapest antibiotics hence widely used. The challenge is that most farmers cannot afford alternative, relatively more expensive drugs. This coupled with low awareness on AMU and AMR is likely to further exacerbate the burden of AMR in the region. This review has revealed high prevalence of AMR, including MDR bacteria in the environment, probably associated with anthropogenic activities, application of manure in farming activities and agricultural wastes from community [61, 69, 85, 86]. There is potential spillage of resistant isolates from sewage, humans, companion and domestic animals and industries to the environment [69, 87].

A few studies compared AMR genes in humans, animals and environment and detected *bla*_CTX-M_, *bla*_TEM_, *bla*_SHV,_ *bla*_OXA,_ *aac(6*′*)- Ib-cr, tet(A)*, *tet(B), sul1*, *sul2,* and *qnr* in all the three compartments [64–67] This finding seems to suggest that these MDR pathogens have high propensity to spread widely and cause infections that are difficult treat [18, 68, 88]. Due to limited laboratory capacity in most of the countries the identified AMR genes could just be a tip of iceberg, representing only a fraction of transmitted genes. This calls for more support to these countries to establish systematic AMR surveillance using more advanced techniques such as Whole Genome Sequencing for better understanding the magnitude, spread and evolution of MDR pathogens [67, 89]. Such information is critical in planning effective interventional measures.

The findings indicate that currently most countries in Africa do not have AMU and AMR surveillance systems and are at different stage of developing them. It may takes a long while before implementation. The different approaches used in different countries leads to inconsistency results which are difficult to compare at national, regional and international perspectives [86]. The results reported do not conform to the international reporting systems since there is no appropriate system for data collection, identifications, coordination and reporting [90]. It is important for countries in Africa to adopt internationally recommended surveillance systems that will take into account data collected from animal, agriculture, environment and human sectors. This review suggests that African countries should seek support from global AMR network to be assisted to develop and implement AMR surveillance under one health approach.

It is important to highlight a number of limitations that were uncovered during the review. First of all, information on AMU is based on percentage of farms using antimicrobial agents rather than the Defined Daily Doses Animals as recommended by the World Health Organization. Furthermore, most of the AMR studies were based on phenotypic rather than molecular techniques, thus limiting understanding of transmission dynamics. In addition, lack of standardized laboratory protocol may account for variations in the level of AMR reported within and between countries. More importantly, there is limited One Health approach surveillance of AMR; hence different sectors employ different approaches in monitoring AMU and AMR. Finally, weak enforcement of the available regulations leads to unmonitored production, distribution, handling, storage and sale of veterinary drugs. It is important that these limitations are taken into consideration as countries in the region continue to plan, develop, refine and implement their National One Health AMR plans.

## Conclusion

There are very high levels of antimicrobial use and antimicrobial resistance, especially for tetracycline, aminoglycoside and penicillin in animal production systems in Africa. This is likely to escalate the already high prevalence of antimicrobial resistance and multi drug resistance in the continent. This, coupled with weak regulations and antimicrobial resistance surveillance systems in the region is a great concern to the animals, environment and humans as well. It is important that African countries strengthen their respective AMU and AMR regulations and surveillance systems to address the challenges identified in this review.

## Supplementary information

**Additional file 1.** Data generated.

## Abbreviations

AGISAR: Advisory Group on Integrated Surveillance of Antimicrobial Resistance
AMR: Antimicrobial resistance
AMU: Antimicrobial use
FAO: Food and Agriculture Organization
ILRI: International Livestock Research Institute
MDR: Multi drug resistance
OIE: Office International des Epizooties
WHO: World Health Organization
