# Supplementary material for: Antimicrobial use and resistance in food-producing animals and the environment: an African perspective
Source: Antimicrob Resist Infect Control. 2020 Mar 3;9:37. doi: 10.1186/s13756-020-0697-x (PMC7053060; doi:10.1186/s13756-020-0697-x)
Supplement: Supplementary file 1 — Additional file 1. Data generated. [file 13756_2020_697_MOESM1_ESM.docx]

**DATA GENERATED**

**A: RESEARCH ARTICLES**

| **COUNTRY** | **AUTHOR** | **SAMPLE** | **ORGANISM** | **% AMU** | **%AMR** | **%MDR** |
| --- | --- | --- | --- | --- | --- | --- |
| Tanzania | Hamis et al. 2012 | Poultry | *E. coli* |  |  | 92.60% |
| Tanzania | Moremi et al. 2016 | Fish and muddy water | *E. coli and K. pneomoniae* |  | 100 |  |
| Tanzania | Katakweba et al. 2016 | Pigs and Human | *S. aureus* |  | 4 |  |
| Ghana | Boamah et al. 2016 | Poultry |  | 98% |  |  |
| Ghana | Donkor et al. 2012 | Food animals | *E. coli* | 98% |  | 92% |
| Tanzania | Katakweba et al. 2012 | Food animals |  | 100% |  |  |
| South Africa | Eager et al. 2012 | Food animals |  |  |  |  |
| Cameroon | Kamini et al. 2016 | Poultry |  | 100% |  |  |
| Sudan | Sirdar et al 2012 | Poultry |  | 92.50% |  |  |
| Ethiopia | Atnafie et al. 2017 | Cattle, human &animal products | *E.coli* |  | 4.70% |  |
| Kenya | Odwar et al. 2014 | Poultry | *E.coli* |  | 75% | 42.90% |
| Zambia | Chishimba et al. 2015 | Poultry | *E.coli* |  |  | 85.70% |
| Tunisia | Soufi et al. 2009 | Poultry | *E.coli* |  |  | 96% |
| Nigeria | Adesokan et al. 2015 | Food animals |  | 77.50% |  |  |
| Zimbabwe | Khumalo et al. 2014 | Food animals | *salmonella* |  |  | 14.2-50% |
| Zambia | Mainda et al. 2015 | Cattle | *E.coli* |  | 98.67% |  |
| Uganda | Afema et al. 2016 | Effluent & wsp, Poultry and Pigs | *salmonella* |  | 39.70% |  |
| Zambia | Mubita et al. 2008 | Cattle | *E.coli/ Enterococci* | 100% |  | 100% |
| Nigeria | Fashae et al. 2011 | Poultry and Pigs | *E.coli* |  |  | 20% |
| Kenya | Christabel et al. 2012 | Water, soil, veg, meat | *E.coli, Salmonella and Shigella* |  |  | 18.75% |
| Ghana | Rasmussen et al. 2015 | Poultry | *E.coli* |  |  | 56.90% |
| Tanzania | Caudel et al. 2017 | Food animals |  | 74% |  |  |
| Ghana | Sekyere 2014 | Food animals |  | 100% |  |  |
| Tanzania | Kimera et al.2015 | Cattle |  | 85% |  |  |
| Tanzania | Nonga et al. 2009 | Poultry |  | 90% |  |  |
| Sudan | Elytaibet al. 2012 | Food animals |  | 95% |  |  |
| South Africa | Adegoke and Okoh 2014 | Pigs, Cattle, goat | *Staphylococcus* |  |  | 68% |
| South Africa | Okoh and Igbinosa 2010 | Effluents | *Vibrio spp* |  |  | 100% |
| Algeria | Dib et al. 2018 | Fish | *E.coli and Salmonella* |  |  | 100% |
| Tanzania | Lupindu et al.2015 | Cattle, human, soil, &water | *E.coli* |  | 42%, 34%,18%, 6% (C-H-S-W) |  |
| Nigeria | Ayandiran et al. 2018 | Poultry | *E.coli* |  | 49% |  |
| Egypt | Hamza et al. 2016 | Poultry, water | *K. pneumoniae* |  |  | 43% |
| Ethiopia | Eguale et al. 2017 | Cattle, Pigs, Poultry | *salmonella* |  |  | 58.60% |
| Kenya | Onyango et al. 2014 | Pigs | *salmonella* |  |  | 45.30% |
| South Africa | Iweriebor et al. 2015 | Cattle | *E.coli* |  |  | 100.00% |
| South Africa | Madoroba et al. 2017 | Cattle | *salmonella* |  |  | 71.70% |
| Tunisia | Ben Said et al. 2015 | Waste and surface water | *E.coli, K.pneumoniae, Citrobacter* |  |  | 100% |
| Tunisia | Tahran et al. 2015 | Waste water | *Pseudomonas,Acinetobacter, Exiguobacterium, Delftia and Morganella* |  |  | 100% |
| Nigeria | Adesoji et al. 2015 | Water | *pseudomonas* |  |  | 95.40% |
| Nigeria | Ngbede et al. 2017 | Veg, soil, Poultry, Cattle, manure | *Enterococci* |  | 94.60% |  |
| Ethiopia | Tufa et al. 2018 | Food animals |  | 85% |  |  |
| Angola | Ribeiro et al. 2016 | Cattle, Pigs, Poultry, waste water, goat, human and feeds | *E.coli* |  | 50% |  |
| Tunisia | Gharsa et al. 2012 | Donkey | *S. aureus* |  | 40% |  |
| Tunisia | Gharsa et al. 2012 | Sheep | *S. aureus* |  |  |  |
| Tunisia | Gharsa et al. 2015 | Cattle, goats |  |  | 7% |  |
| South Africa | Schellack et al. 2017 | Food animals and environment |  |  |  |  |
| Cameroon | Founou et al. 2018 | Pigs | *K. pneumoniae* |  | 22% |  |
| Cameroon, South Africa | Founou et al. 2018 | Pigs and human | *Staphylococcus* |  |  | 85% |
| Nigeria | Ojo et al. 2015 | Poultry | *E.coli* |  | 100% |  |
| Egypt | Braun et al. 2016 | Cattle | *E.coli* |  | 42.80% |  |
| Nigeria | Fashae et al. 2010 | Poultry | *E.coli* |  |  | 62% |
| Tunisia | Ben Sallem et al. 2014 | Cattle, Poultry, human | *E.coli* |  |  |  |
| Tunisia | Gharbi et al. 2018 | Poultry | *Campylobacter* |  | 100 |  |
| Algeria | Belmahdi et al. 2016 | Poultry | *E.coli* |  |  | 90% |
| South Africa | Ateb aand Bezuidenhout. 2008 | Cattle and Pigs | *E.coli* |  |  | 93.40% |
| Nigeria | Adenipekun et al. 2015 | Cattle, Poultry and Pigs | *E.coli* |  | 92.90% | 45.50% |
| Nigeria | Adelowo et al. 2014 | Poultry | *E.coli* |  |  | 83% |
| South Africa | Bester and Essack. 2008 | Poultry | *Campylobacter* |  |  | 66% |
| Egypt | Ahmed and Shimamoto. 2012 | Poultry | *salmonella* |  |  | 81% |
| Egypt | Hussein et al. 2013 | Poultry | *E.coli* |  |  | 80% |
| South Africa | Oguttu et al. 2008 | Poultry | *E.coli* |  |  |  |
| Egypt | Elhariri et al. 2017 | Camel | *Pseudomonas aerognosa* |  | 45% | 73% |
| Algeria | Djeffal et al. 2017 | Poultry, human | *salmonella* |  | 51.10% |  |
| Tunisia | Kilani et al. 2015 | Poultry | *E coli* |  | 26.10% |  |
| Egypt | Shazily et al. 2017 | Poultry | *E coli* |  | 82.50% |  |
| Nigeria | Beshiru et al. 2017 | Pigs | *enteroccocus* |  | 48% |  |
| South Africa | Igwaran et al. 2018 | Waste water plant | *E.coli* |  | 75.9 avrg |  |
| South Africa | Olaniran et al. 2015 | Treated waste water &surface water | *Listeria &Aeromonas* |  | 24.36(L)/29A |  |
| South Africa | Abia et al. 2015 | Sediments | *E.coli* |  |  | 84% |
| Nigeria | Alhaji et al. 2018 | Poultry |  | 88.50% |  |  |
| Ghana | Andoh et al. 2016 | Poultry | *Salmonella* |  |  | 40.40% |
| Burkinafaso | Kagambega et al. 2013 | Cattle, Poultry, Pigs | *Salmonella* |  | 14.00% |  |
| Zimbabwe | Saidi et al. 2013 | Poultry | *E.coli* |  |  | 100% |
| Ethiopia | Abera et al. 2014 | Water | *E coli, P.aeroginosa* |  |  | 66.70% |
| South Africa | Dlamini et al. 2018 | Cattle | *Salmonella* |  |  | 94.30% |
| South Africa | Igbinosa. 2015 | Cattle, goats | *Salmonella* |  |  | 100 |
| Tanzania | Katakweba et al. 2017 | Cattle, Pigs, Poultry | *E.coli* |  |  | 65.10% |
| Nigeria | Chah et al. 2018 | Chicken | *E.coli, K.pneumoniae, Enterobacter* |  |  | 40% |
| South Africa | Ebomah et al. 2018 | River water | *E.coli* |  |  | 21.90% |
| Uganda | Ikwap et al. 2014 | Pigs | *Salmonella* | 40.6 |  | 57% |
| South Africa | Adefisoye and Okoh. 2015 | Treated waste water | *E.coli* |  |  | 32.70% |
| South Africa | Adefisoye and Okoh. 2017 | Treated waste water | *Vibrio spp* |  |  | 81.00% |
| South Africa | Mkize et al. 2017 | Poultry | *Staphylococcus* |  |  | 100% |
| South Africa | Zishiri et al. 2016 | Poultry | *Salmonella* |  |  | 100% |
| Tunisia | Ben Salem et al. 2017 | Poultry | *Salmonella* |  |  | 33.80% |
| Ethiopia | Beyene et al. 2017 | Cattle | *Staphylococcus* |  |  | 100% |
| Ethiopia | Moges et al. 2014 | Waste water treatment ponds | *K.pneumoniae* |  | 54.20% |  |
| Nigeria | Beshiru et al. 2016 | Pigs | *E.coli* |  |  | 80% |
| Algeria | Brahmi et al. 2017 | Fish | *E.coli* |  |  | 54.50% |
| Kenya | Adelaide and Okemo. 2008 | Poultry | *E.coli* |  |  | 67.20% |
| Morocco | Oubrim et al. 2012 | Treated waste water | *Salmonella* |  |  | 45% |
| Morocco | Ennaji et al. 2008 | Poultry | *Listeria* |  |  |  |
| Tunisia | Abbassi et al. 2017 | Poultry, bovine and ovine | *E.coli* |  |  | 44.20% |
| Algeria | Bouzidi et al.2012 | Poultry | *Salmonella* |  |  | 46% |
| Morocco | Khallaf et al. 2014 | Poultry meat | *Salmonella* |  | 65.78% | 42.10% |
| Algeria | Anssour et al.2016 | Treated waste water | *Enterobacteriaceae* |  | 88.20% |  |
| Algeria | Alouache et al. 2013 | Treated waste water | *E.coli/K.pneomoniae* |  |  | 85% |
| Algeria | Habi and Daba. 2009 | Treated waste water | *Enterobacteriaceae* |  |  |  |
| Algeria | Tafoukt et al. 2017 | River water | *Enterobacteriaceae* |  |  | 100% |
| Bernin | Mousse et al. 2015 | Vegetables | *E.coli* |  |  |  |
| Nigeria | Beshiru et al. 2016 | Pigs | *E.coli* |  |  | 80% |
| Benin | Boko et al. 2013 | Guinea fowls | *Salmonella* |  |  |  |
| Botswana | Gaedirelwe and Sebunya. 2008 | Poultry | *Salmonella* |  |  |  |
| Burkinafaso | Kagambega et al. 2018 | Poultry and human | *Salmonella* |  |  | 100% |
| Ethiopia | Abunna et al. 2016 | Poultry | *Salmonella* |  |  | 94.70% |
| Cameroon | Akoachere et al. 2009 | Pigs | *Salmonella* |  |  | 50.70% |
| Ethiopia | Addis et al. 2011 | Cattle | *Salmonella* |  |  | 83% |
| Cameroon | Wadoum et al. 2016 | Poultry | *Salmonella, E.coli, Listeria, Clostridium, Staphylococcus* | 80% |  | 100% |
| Cameroon | Akoachere et al. 2013 | Water | *V.cholerae* |  |  | 92% |
| Chad | Tabo et al. 2013 | Poultry | *Salmonella* |  | 33.30% |  |
| Egypt | Osman and Elhariri. 2013 | Poultry | *C. perfringes* |  |  | 100% |
| Egypt | Ahmed et al. 2009 | Cattle | *Salmonella* |  |  | 66.70% |
| Egypt | Dahshan et al. 2015 | Poultry | *E.coli* |  |  | 80% |
| Egypt | El‑Sharkawy et al. 2017 | Poultry | *Salmonella* |  |  |  |
| Egypt | Osman et al. 2014 | Fish | *Enterococcus* |  | 100% |  |
| Egypt | Abdel-Maksoud et al. 2015 | Poultry | *S. enterica* |  |  | 82% |
| Egypt | Abo-State et al. 2012 | River water | *E.coli* |  | 100% | 82.50% |
| Ethiopia | Bekele and Ashenafi. 2010 | Cattle, Poultry | *Enteroccocus/Salmonella* |  |  | 80% |
| Ethiopia | Kassa et al. 2007 | Cattle, Poultry, Pigs, sheep | *Campylobacter* |  |  | 14.50% |
| Ethiopia | Ewnetu and Mihret. 2010 | Poultry and human | *Campylobacter* |  | 25% |  |
| Ethiopia | Eguale et al. 2016 | Cattle | *Salmonella* |  | 100% | 70% |
| Kenya | Kikuvi et al. 2010 | Pigs | *Salmonella* |  |  | 7.10% |
| Ethiopia | Molla et al. 2006 | Sheep, goats | *Salmonella* |  |  | 31.80% |
| Ethiopia | Fekadu et al. 2015 | Treated waste water | *Salmonella* |  |  |  |
| Ghana | Sackey et al.2001 | Poultry | *Campylobacter, Salmonella, Shigella, E.coli* | | | 100% |
| Ghana | Karikari et al. 2017 | Cattle, Goats, Pigs, Sheep | *Campylobacter* |  |  | 66.60% |
| Ghana | Adzitey et al. 2015 | Water | *E.coli* |  | 37.90% | 37.50% |
| Kenya | Wawire et al. 2013 | Water, Fish, Goat, Poultry, Donkey, Cattle | *Enterobacteriaceae* |  | 53.80% |  |
| Kenya | Kikuvi et al. 2007 | Cattle, Pigs, Poultry | *Salmonella* |  | 35.70% | 7.10% |
| Morocco | Bennani et al. 2012 | Sediments, ocean water, shelfish | *Enteroccocus* |  | 64.70% | 13.60% |
| Senegal | Cardinale et al. 2005 | Poultry, human | *Campylobacter* |  | 11.10% |  |
| Egypt | Hassanain. 2011 | Cattle, buffalo, sheep, Poultry, human | *Campylobacter* |  | not stated |  |
| Senegal | Dione et al. 2009 | Poultry | *Salmonella* |  |  | 77.70% |
| Sudan | Elmadiena et al. 2013 | Fish, Poultry, Cattle, water, human | *Salmonella* |  | 98.40% | 71.90% |
| Sudan | Fadlalla et al. 2012 | Cattle, Poultry, camel, human | *Salmonella* |  | 80.67% | 37.82% |
| Uganda | Kateete et al. 2013 | Cattle, human | *Enterococcus* |  | 100% |  |
| Tunisia | Klibi et al. 2014 | Poultry, Cattle, sheep | *Enterococcus* |  |  | 18.50% |
| Tunisia | Turki et al. 2012 | manure, human, soil, treated waste water, animals | *Salmonella* |  |  | 5.30% |
| Tunisia | Mnif et al. 2012 | Poultry | *E.coli* |  |  | 100% |
| Tunisia | Maamar et al. 2012 | Poultry | *E.coli* |  | 100% |  |
| Tunisia | Ben saidi et al. 2016 | Vegetable, soil and irrigation water | *Enterococcus* |  |  | 100% |
| Tunisia | Sarra et al. 2013 | Fish | *Enterococcus* |  | 100% |  |
| Tunisia | Saidi et al. 2013 | Fish, sea and waste water | *Aeromonas hydrophila* |  |  | 74.19% |
| Tunisia | Ben saidi 2016 | Sea food | *Enterococcus* |  |  | 31.80% |
| Uganda | Wamala et al. 2016 | Fish | *Aeromonas* |  | 100% |  |
| Uganda | Sasanya et al. 2005 | Poultry |  | 96.70% |  |  |
| Uganda | Bosco et al. 2012 | Cattle, Poultry, Pigs, human | *Salmonella* |  | 54% |  |
| Uganda | Majalija et al. 2010 | Poultry | *E.coli* |  | 87% | 65% |
| Uganda | Kasozi et al. 2014 | Cattle | *S.aureus* |  |  | 71.40% |
| Nigeria | Olowe et al. 2015 | Cattle and Pigs | *E.coli* |  |  | 63.20% |
| Tanzania | Mwaikono et al. 2015 | Dump site garbage | *Enteric bacteria* |  |  | 56% |
| Zambia | Sakala. 2017 | Fish | *Lactococcus, Streptococcus, Aeromonas* |  |  | 100% |
| Algeria | Messad et al. 2014 | Poultry | *Campylobacter* |  | 100% | 100% |
| Zimbabwe | Simango and Mwakurudza. 2008 | Poultry | *Clostridium deficile* |  | 100% |  |
| Zimbabwe | Makaya et al. 2012 | Poultry | *Salmonella* |  | 26% | 12.10% |
| Zimbabwe | Siwela et al. 2007 | Poultry | *E.coli* |  | 100% |  |
| Zimbabwe | Simago. 2013 | Poultry, human | *Campylobacter* |  | (50%-h)/(82%-P) | |
| Zimbabwe | Mercat et al. 2016 | Cattle, buffalo | *E.coli* |  | 100% |  |
| DRP Congo | Boeck et al. 2012 | Treated waste water, river water | *K.pneumoniae, E.cloacae* | | 100% |  |
| Nigeria | Alhaji and Isola. 2018 | Cattle, sheep and goats |  | 100% |  |  |
| Botswana | Sara et al 2015 | Domestic &Wild life | *E.coli* |  | 13.30% |  |
| South africa | Chipangura et al 2017 | Small animals (Canine), human | | 91.16% |  |  |
| Nigeria | Iwenzaura 2017 | Hospital environment/Cattle | *E.coli* |  |  |  |
| Botswana | Jobbins and Alexander. 2015 | Domestic and wild animals | *E.coli* |  | 41.30% | 13.30% |
| Egypt | Afifi. 2013 | Environmental surface | *K.pneumoniae, E.coli* |  | 100% |  |
| South Africa | Mathole et al. 2016 | Pigs, goat and Poultry | *Salmonella* |  | 66.70% |  |

**B: REVIEWS, BOOKS, REPORTS, POLICY FORUMS AND PERSPECTIVES**

| **GEOGRAPHIC AREA** | **AUTHOR** | **SPECIES/ SAMPLE** | **ARTICLE TYPE** |
| --- | --- | --- | --- |
| African region | Founou et al. 2016 | Food chain | Review article |
| Developing countries | Ayukekbong et al. 2017 | Human and animal | Review article |
| Africa | Alonso et al. 2017 | Food animals | Review article |
| Tanzania | Seni et al. 2017 | Human, animals and environment | Systematic review |
| South Africa | Moyane et al. 2013 | Food animals | Review article |
| Sub Saharan Africa | Kariuki & Dougan. 2014 | Human and animal | Review article |
| Nigeria | Oloso et al. 2018 | Food animal and environment | Review article |
| Africa | Lozano et al. 2016 | Animals and food | Review article |
| Developing countries | Manyi-Loh et al. 2018 | Agriculture & environment | Review article |
| Africa | Founou et al. 2018 | Food animals | Systematic review |
| Lower and Middle Income Countries | Schar et al. 2018 | Animal production | Policy forum |
| Africa | Essack et al. 2016 | Human , animals and environment | Perspective |
| Developing countries | Sosa et al. 2010 | General | Book |
| Developing countries | Grace. 2015 | Agriculture and animals | Book |
| Global | Hoornweg and Bhada-Tata. 2012 | Environment | Report |
